# A biosynthetically informed distance measure to compare secondary metabolite profiles

**DOI:** 10.1007/s00049-017-0250-4

**Published:** 2017-11-27

**Authors:** Robert R. Junker

**Affiliations:** Department of Ecology and Evolution, University Salzburg, Hellbrunnerstraße 34, 5020 Salzburg, Austria

**Keywords:** Biosynthesis, Enzymes, Pathways, Plant volatiles, Statistics, Volatile organic compounds

## Abstract

Secondary metabolite profiles are one of the most diverse phenotypes of organisms and can consist of a large number of compounds originating from a limited number of biosynthetic pathways. The statistical treatment of such profiles often is complicated due to their diversity as well as the intra- and interspecific variability in the quantitative and qualitative composition of secondary metabolites. Most importantly, the assumption of independence of the presence/absence and the quantity of compounds is violated due to the shared biosynthetic origin of many compounds. Therefore, I propose a biosynthetically informed pairwise distance measure that fully considers the biosynthesis of the compounds and thus quantifies the similarity in the enzymatic equipment of two samples. The biosynthetic similarity of compounds is calculated based on the proportion of shared enzymes that are required for their biosynthesis. Using this information (provided as dendrogram structure) and the quantitative composition of the samples, generalized UniFrac distances are calculated measuring that fraction of the dendrogram (i.e., the branch lengths) that is unique to either of the samples but not shared by both samples. To allow a straightforward cross-platform application of the approach, I provide functions for the statistical software R and sample data sets. A hypothetical and a real world example show the feasibility of the biosynthetically informed distances *d*_*A,B*_ and highlight the differences to conventional distance measures. The advantages of this approach and potential fields of application are discussed.

**Electronic supplementary material:**

The online version of this article (10.1007/s00049-017-0250-4) contains supplementary material, which is available to authorized users.

## Introduction

Organisms synthesize a plethora of secondary metabolites that are of physiological and ecological importance (Dudareva et al. 2013; Steiger et al. 2011). Species may produce few to thousands of compounds originating from various biosynthetic origins (Wink 2010; Wyatt 2014). A single plant species, for example, may emit volatiles originating from the shikimate (aromatic compounds), the lipoxygenase (LOX, fatty acid-derived compounds), the 2-*C*-methyl-d-erythritol 4-phosphate (MEP, monoterpenes, diterpene-derived compounds, and tetraterpene-derived compounds), and the mevalonate pathway (MVA, sesquiterpenes and sesquiterpene-derived compounds) (Dudareva et al. 2013; Knudsen et al. 2006). Each of these pathways feature a backbone of enzymes producing the precursors for the final products that are subsequently produced by the final enzymes of the respective pathways (Dudareva et al. 2013). Terpenoid compounds, for instance, produced by just two pathways are enormously diversified both within species and across plant lineages (Pichersky and Raguso 2016). This high diversity of secondary metabolites results in a pronounced intra- and interspecific variation in the qualitative and quantitative composition of secondary metabolite profiles (Junker 2016; Junker et al. 2017; Kuppler et al. 2016; Masclaux-Daubresse et al. 2014; Knudsen et al. 2006). As a result of the diversity and variability of secondary metabolites, species synthesize a unique composition of compounds, which means that only a relatively small proportion of compounds can be detected in more than one or few species sampled in a common environment (Junker 2016).

The diversity of secondary metabolites originating from a limited number of pathways and the consequential high number of singletons in data sets (compounds occurring only in one sample) introduce some difficulties in the statistical treatment of secondary metabolite profiles (Junker and Parachnowitsch 2015; Brückner and Heethoff 2017). First, data sets containing the composition of secondary metabolites (of many species) are usually zero-inflated. Second, due to the shared biosynthetic origin, the identity and quantity of a compound is not independent of the other compounds in the same sample. Accordingly, it has been shown that the biosynthesis of the compounds explains a large proportion of the quantitative composition of plant volatiles (Junker et al. 2017). One of the most commonly applied approaches to statistically compare compositions is the calculation of distances of relative or absolute quantities between samples (often Bray–Curtis dissimilarities or Euclidean distances) followed by non-metric multidimensional scaling (Brückner and Heethoff 2017; Legendre and Legendre 2012). Both mentioned distance/dissimilarity measures, however, do not consider the biosynthetic origin of the compounds but treat each compound as independent variable. Euclidean distances are strongly affected by the large number of zeros in the data often leading to high similarities between samples that have in common that they both do not comprise a number of compounds, while they may not share the presence of the relevant compounds. Bray–Curtis dissimilarities, in contrast, ignore compounds that are not present in a sample pair and thus this distance measure is preferable compared to Euclidean distances. Nonetheless, a large variation in the quantity of compounds may also lead to large distances despite a very similar or the same qualitative composition. Other commonly applied distance measures face similar problems (Legendre and Legendre 2012). While some of the problems of distance measures can be avoided by choosing the most suitable for the research question or by transforming the quantitative data in meaningful way, none of the available distance measures, to the best of my knowledge, considers the biosynthetic origin of the compounds and thus their dependence on each other.

To provide a statistical approach that calculates distances that consider the biosynthesis of compounds between samples, I introduce a biosynthetically informed distance measure *d*_*A,B*_. This biosynthetically informed distance measure *d*_*A,B*_ integrates the quantitative compositions of secondary metabolites in samples *A* and *B* with the biosynthetic similarity of compounds present in samples *A* and *B* based on the number of shared enzymes.

### A biosynthetically informed distance measure

**Fig. 1:**
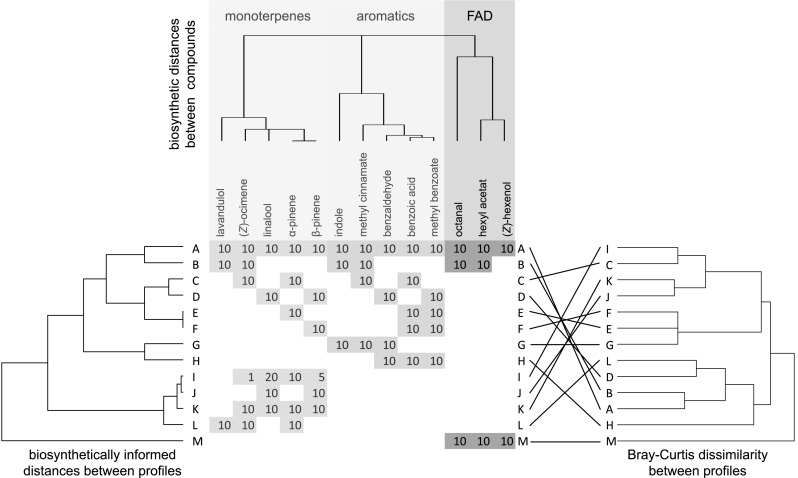
Hypothetical secondary metabolite profiles A–M. Profiles are composed of monoterpenes, aromatics, and/or fatty acid derivatives (FAD). Biosynthetic distances of compounds based on the number of shared enzymes are visualized by the upper dendrogram. Numbers in rows are the quantities of the compounds in each of the profiles, no number means that this compound is absent in the profile. Similarities between profiles based on biosynthetically informed distances *d*_*A,B*_ are visualized in the left dendrogram, similarities based on Bray–Curtis dissimilarities in the right dendrogram. Differences between dendrograms are highlighted as tanglegram. Major differences are listed and explained in Table 1

The biosynthetically informed distance measure *d*_*A,B*_ combines data on the enzymes involved in the biosynthesis of secondary metabolites and the quantitative composition of secondary metabolites. In the following, the information on the quantitative (or qualitative) composition of compounds as well as the biosynthesis of the compounds is used to calculate UniFrac distances, which has been developed to compute differences between microbial communities (Lozupone and Knight 2005). The data structure of site x microbial species matrices (with sites as rows and microbial species (operational taxonomic units OTUs) as columns, entries are abundances per OTU and site) obtained from high throughput techniques is similar to the one observed in sample × secondary metabolite matrices (with samples as rows and secondary metabolites as columns, entries are quantities per metabolite and sample). Both are often zero-inflated and information about the (phylogenetic or biosynthetic) similarity of the species or secondary metabolite can add valuable information to the distance between sites or samples. Instead of directly considering microbial taxa, UniFrac distances are based on the phylogeny describing the evolutionary similarity of taxa. UniFrac calculates the fraction of the branches (i.e., their lengths) of a phylogenetic tree that are unique to one of the samples but not shared by taxa present in both samples. Thus, not the identity of a taxon is considered but its evolutionary history. This approach can be adopted for secondary metabolites that share parts of their biosynthetic pathway. Here the biosynthetic information allows evaluating the similarity in the enzymatic equipment of an organism. Thus, the interdependence between compounds is fully considered and the distance between samples is calculated based on their biosynthetic abilities and not exclusively by the number of shared compounds. This means that samples that are dominated by compounds that originate from the same biosynthetic pathway but do not share a single compound are still more similar to each other than two samples that feature compounds of different pathways (Fig. 1). Therefore, in contrast to conventional distance/dissimilarity measures (e.g., Bray–Curtis), the biosynthetically informed distance measure *d*_*A,B*_ is the first one that incorporates biosynthetic pathways into the quantification of distances between compositions of secondary metabolites. In an ecological context where, for example, animals respond to individual compounds and not to pathways, the biosynthetically informed distance measure *d*_*A,B*_ may be irrelevant. However, for research questions on a phylogenetic signal in secondary metabolite profiles across taxon lineages, on chemotaxonomy separating species based on the composition of secondary metabolites, or on the functional diversity of ecological communities, the biosynthetically informed distance measure *d*_*A,B*_ adds a fundamentally new information and will lead to novel insights into the evolution and ecology of secondary metabolites.

## Sketch of the approach

The biosynthetically informed distance measure *d*_*A,B*_ is calculated combining a number of pre-existing R packages as well as information on the biosynthetic pathways of secondary metabolites. To allow a straightforward cross-platform application of the approach, I provide functions for R (R Core Team 2016) and sample data sets in electronic supplementary material 1.

### Data requirement

Two data sets are required to calculate biosynthetically informed distance measure *d*_*A,B*_:

1. A matrix with samples as rows, secondary metabolites as columns and information on the quantity of each compound in each sample as entries. Qualitative information (the presence or absence of a compound in a sample) is sufficient.
2. A presence/absence matrix with secondary metabolites as rows and enzymes as columns. This matrix contains information about the enzymes involved in the biosynthesis of each compound. Such a matrix had been compiled for 150 plant volatiles and can be downloaded as supporting information S3 in Junker et al. (2017).

### Approach

Using these data sets, few statistical steps need to be performed to obtain the biosynthetically informed distance *d*_*A,B*_ between samples *A* and *B*.

1. Calculation of Sørensen dissimilarities between the secondary metabolites based on the number of shared enzymes using the R package *vegan* (Dixon 2003). Large Sørensen dissimilarities indicate that secondary metabolites require completely different or largely non-overlapping sets of enzymes in their biosynthesis, whereas small distances indicate a large number of shared enzymes that are involved in their biosynthesis. Note that some compounds have Sørensen dissimilarities = 0 in the case of multi-product enzymes (e.g., α- and β-pinene that are synthesized by the same terpene synthase,see Fig. 1).
2. Based on the Sørensen dissimilarities matrix of the compounds, a hierarchical cluster analysis is performed implemented in the R package *stats* (R Core Team 2016). The resulting clustering tree is then converted into an object of the class “*phylo*”, which is required by the following step.
3. Using the converted clustering tree containing the information on the biosynthetic similarity and the matrix containing the composition of secondary metabolites of the samples, weighted generalized UniFrac distances between all sample pairs are calculated using the R package GUniFrac (Chen et al. 2012). UniFrac distances require the dendrogram information from the previous step (2) and quantify the fraction of the total branch length of the dendrogram that leads to compounds present in one sample or the other, but not both (Lozupone and Knight 2005). Weighted generalized UniFrac have the advantage that neither to much weight is assigned to compounds in low nor in high quantities (Chen et al. 2012). Weighted UniFrac distances are used as biosynthetically informed distance *d*_*A,B*_ that are weighted by the quantity of the compounds.

To facilitate an easy calculation of biosynthetically informed distance *d*_*A,B*_, steps 1–3 are compiled in a single function *BioSynDist()*, which is provided as source file in electronic supplementary material 1. The function *BioSynDist()* uses default settings of all functions described in steps 1–3, which can be changed in the source function if desired.

#### Alternative approach requiring less detailed information on biosynthesis of compounds

Biosynthetically informed distances as described above require detailed information on the enzymatic pathways of compound biosynthesis. This information, however, may not always be available preventing the calculation of biosynthetically informed distances. To allow a more general application of biosynthetically informed distances, I am proposing an additional method that requires only the knowledge about the chemical classes of the compounds instead of the enzymatic pathways. Alternatively, any other information on the characteristics of the compounds (functional groups, chain length of cuticular hydrocarbons, etc.) can be used in this approach. The potentially lower resolution of the approach relying on chemical classes or other characteristics instead of enzymes can be compensated by merging the biosynthetically informed distances with Bray–Curtis dissimilarities, which results in astonishingly similar distances compared to those obtained from trials using information of the enzymes involved in the synthesis of the compounds (see below). To allow a straightforward cross-platform application of this alternative approach, I provide functions for R (R Core Team 2016) and sample data sets in supporting information 1.

### Data requirement for the alternative approach

Two data sets are required to calculate biosynthetically informed distance measure *d*_*A,B*_:

1. A matrix with samples as rows, secondary metabolites as columns and information on the quantity of each compound in each sample as entries. Qualitative information (the presence or absence of a compound in a sample) is sufficient (same dataset as for the main approach).
2. The presence/absence matrix with secondary metabolites as rows and chemical classes or other characteristics of the compounds as columns.

### Alternative approach

Using these data sets, statistical steps 1–3 described above for the main approach have to be performed. The weighted UniFrac distances (step 3) are used as biosynthetically informed distances *d*_*A,B*_ that are weighted by the quantity of the compounds. In the alternative approach, these distances have a very low resolution both regarding the biosynthesis (all compounds of a chemical class are considered to have the same enzymatic pathway) and—because of the utilization of weighted generalized UniFrac distances—the identity of compounds is not considered in this approach. To compensate for this low resolution, biosynthetically informed distance *d*_*A,B*_ are now merged with Bray–Curtis dissimilarities calculated based on the quantitative composition of the secondary metabolite profiles. To merge biosynthetically informed distance *d*_*A,B*_ and Bray–Curtis dissimilarities, the weighted mean of both pairwise distances *mDist* is calculated using *w* (0 ≤ *w* ≤ 1) as weight:

$$mDist=w \times BioSynDistStand+(1 - w) \times DistStand.$$

If *w* is set to 1 *mDist* = *d*_*A,B*_ based on chemical classes (*BioSynDistStand*), if *w* is set to 0 *mDist* = Bray–Curtis dissimilarities (*DistStand*). Both distance measures are standardized between 0 and 1 (*d′* = *d*/max(*d*)) prior to the this step. Values of weight *w* between 0 and 1 merge biosynthetically informed distance *d*_*A,B*_ and Bray–Curtis dissimilarities in different ratios.

To facilitate an easy calculation of biosynthetically informed distance *d*_*A,B*_ based on chemical classes or any other information on the compounds and to merge these distances with Bray–Curtis dissimilarities, steps 1–3 are compiled in a single function *BioSynDist()*, which is provided as source file in supporting information 1. The function *BioSynDist()* uses default settings of all functions described in steps 1–3, which can be changed in the source function if desired. The final step where biosynthetically informed distances *d*_*A,B*_ are merged with Bray–Curtis dissimilarities is compiled in a function *MergeDist()*, which is provided as source file in supporting information 1.

## Results and discussion

To demonstrate the potential of biosynthetically informed distance *d*_*A,B*_, I tested the approach using an hypothetical dataset and a real world example containing samples of floral scent emissions of two plant species. Data on enzymes involved in the biosynthesis of the selected compounds of both examples were obtained from Junker et al. (2017, supporting information S3).

### Hypothetical compositions of secondary metabolites

I generated 13 samples composed of up to 13 secondary metabolites originating from the MEP (five monoterpenes), shikimate (five aromatics), and LOX pathway (three fatty acid derivatives, Fig. 1). The composition of the samples was chosen to highlight the differences of the biosynthetically informed distances *d*_*A,B*_ to conventional distance measures (e.g., Bray–Curtis dissimilarities). Analysis of data presented in Fig. 1 can be replicated using the data sets and R code in electronic supplementary material 1.

**Table 1:** Examples of differences between the biosynthetically informed distance *d*_*A,B*_ and Bray–Curtis dissimilarities as shown in Fig. 1

Profiles	Biosynthetically informed distances	Bray–Curtis dissimilarity
C, D	< (monoterpenes and aromatics present in C and D share a large number of enzymes, respectively)	> (C and D do not share a single compound)
E, F	< (α- and β-pinene are synthesized by the same terpene synthase)	> (only two thirds of the compounds are shared)
G, H	< (aromatics share a large number of enzymes)	> (only one third of the compounds are shared by the bouquets)
I, J, K, L	< (weighted UniFrac distances consider differences in quantitative composition, biosynthetic similarity of bouquets remains high)	> (quantitative composition and number of shared compounds strongly influences values of distances)

Smaller distances in the former measure than in the latter are indicated by “<”; larger distances in the latter than in the former measure are indicated by “>”. Differences are explained based on statistical and biosynthetic reasons

Overall, hypothetical profiles are well separated based on biosynthetically informed distances *d*_*A,B*_ and on Bray–Curtis dissimilarities (Fig. 1). Although distance matrices based on both distance measures are well correlated (Mantel statistic based on Pearson’s product–moment correlation: *r* = 0.64, *p* < 0.001), a visual inspection of the dendrograms reveals important differences between both distance measures, which are highlighted in Table 1. These examples demonstrate how biosynthetic information affects distances, which may be—depending on the research question—a relevant addition to conventional distance measures.

**Fig. 2:**
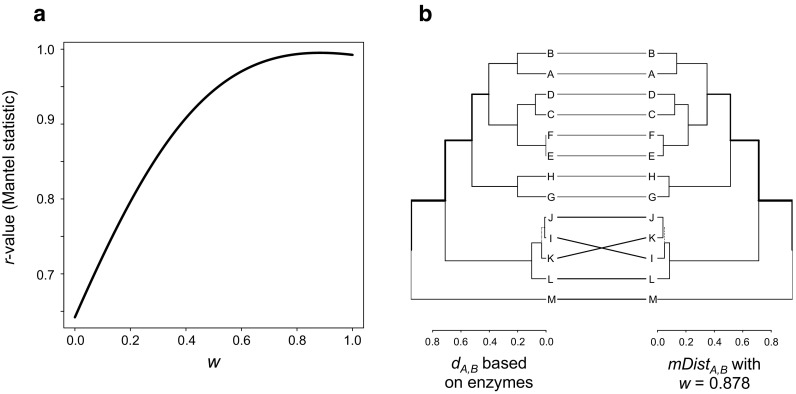
Similarity of biosynthetically informed distance *d*_*A,B*_ based on enzymes and merged distances *mDist*, which is the weighted mean of biosynthetically informed distances *d*_*A,B*_ based on chemical classes and Bray–Curtis dissimilarities. **a** For 0 ≤ *w* ≤ 1, I tested which *w* returns merged distances *mDist* most strongly correlated to biosynthetically informed distance *d*_*A,B*_ based on enzymes. *mDist* calculated with *w* = 0.878 correlated most strongly to *d*_*A,B*_ (*r* = 0.999). **b** Tanglegram visualizing the similarity of biosynthetically informed distance *d*_*A,B*_ based on enzymes and *mDist* (*w* = 0.878). Dendrograms based on both distance measures turned out to be nearly identical

To test whether *mDist* (alternative approach requiring less detailed information on biosynthesis of compounds) reflects biosynthetically informed distance *d*_*A,B*_ based on enzymes and to test which weight *w* returns the best result, I merged biosynthetically informed distance *d*_*A,B*_ based on chemical classes with Bray–Curtis dissimilarities using different weights *w* using the same hypothetical dataset as described above. For each *w*, I tested whether *mDist* correlates to biosynthetically informed distance *d*_*A,B*_ based on enzymes using Mantel statistic based on Pearson’s product–moment correlation. Correlation was highest using *w* = 0.878 (Mantel statistic: *p* < 0.001, *r* = 0.999, Fig. 2). Therefore, *w* = 0.878 is the default setting in the function *MergeDist()*, which, however, is not universally the best setting (as an exercise, the reader may test this using the real world examples (see below) provided in supporting information 1).

This result demonstrates that information on chemical classes or other information on the compounds may be sufficient to obtain results that reassemble those obtained using enzymatic information very well. Although *w* = 0.878 is definitively not the best setting for all data sets, it may be a good value to start with.

### Real world example

**Fig. 3:**
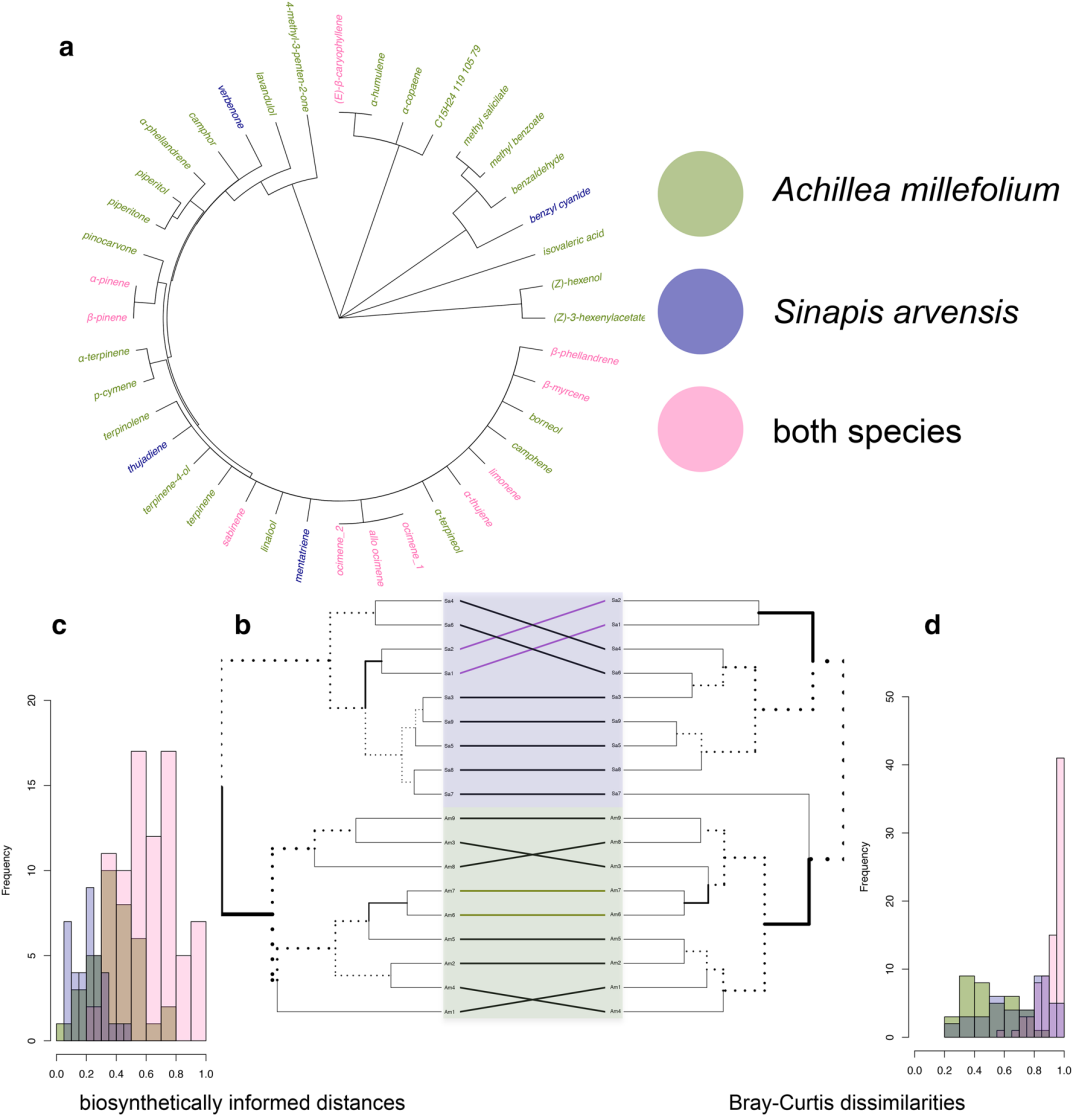
Similarity between floral scent bouquets of *Achillea millefolium* (Am 1–9) and *Sinapis arvensis* (Sa 1–9). **a** Biosynthetic distances of compounds emitted by both plant species based on the number of shared enzymes are visualized by the dendrogram. Compounds exclusively detected in samples of *A. millefolium* are shown in green; those exclusively detected in *S. arvensis* samples in blue, and those emitted by both species in red. **b** Similarities based on biosynthetically informed distance *d*_*A,B*_ (left) and Bray–Curtis dissimilarities (right). **c, d** Histograms show distribution of pairwise distances based on biosynthetically informed distance *d*_*A,B*_ (**c**) and Bray–Curtis dissimilarities (**d**) between the samples of the same species (*A. millefolium* in green, *S. arvensis* in blue) and distances of samples from different species (red). (Color figure online)

I used the floral scent bouquets of *Achillea millefolium* (Asteraceae, Larue et al. 2016) and *Sinapis arvensis* (Brassicaceae, Kuppler et al. 2016) and extracted the biosynthetic information from Junker et al. (Junker et al. 2017, supporting information S3). For both plant species, I included samples from *n* = 9 individuals. Data sets on composition and biosynthetic information are available in electronic supplementary material 1. I used the R package *dendextend* to plot the tanglegram, which helps to compare the dendrogram based on biosynthetically informed distances and the dendrogram based on Bray–Curtis dissimilarities. In total, 40 volatile organic compounds were detected in the floral scent bouquets of *A. millefolium* and *S. arvensis* and both bouquets were dominated by monoterpenes. 25 compounds were exclusively emitted by *A. millefolium* flowers, 4 by *S. arvensis* flowers, and 11 compounds were detected in both bouquets (Fig. 3a). Biosynthetically informed distances *d*_*A,B*_ and Bray–Curtis dissimilarities correlated (Mantel statistic based on Pearson’s product-moment correlation: *r* = 0.55, *p* < 0.001), but differences are revealed by a visual inspection of the tanglegram comparing the results based on both distance measures (Fig. 3b). Both distance measures well separated the species, albeit one *S. arvensis* sample clustered closer to *A. millefolium* samples than to conspecifics in the dendrogram based on Bray–Curtis dissimilarities. In general, within-species pairwise biosynthetically informed distances tended to have stronger contrast to between-species pairwise distances (Fig. 3c) than Bray–Curtis dissimilarities (Fig. 3d). In summary, both distance measures were well suited to cluster samples based on the quantitative composition of floral scent compounds (species factor fitted onto NMDS based on biosynthetically informed distances *d*_*A,B*_: *r*^2^ = 0.66, *p* < 0.001; Bray–Curtis dissimilarities: *r*^2^ = 0.72, *p* < 0.001; R package vegan, Dixon 2003). However, the results differ in the distances within species, which may reveal important information about the biosynthetic causes for intra- and interspecific variation.

### Caveats

Biosynthetically informed distance *d*_*A,B*_ requires a detailed knowledge about the enzymes that are involved in the biosynthesis of the compounds identified in samples. Unfortunately, this information is not available for a majority of compounds and there are no databases compiling such information. In the supporting information S3 in Junker et al. (2017), we provide this information for *n* = 150 volatile organic compounds, which may not be sufficient for many studies. For some of the compounds listed, enzymatic pathways have been described in the literature in detail, for many others at least the final enzyme had to be postulated (Junker et al. 2017), which requires expert knowledge and may prevent a general utilization of biosynthetically informed distance *d*_*A,B*_. However, compounds originating from major biosynthetic pathways share a common backbone of enzymes, which may help to assign and postulate enzymes to compounds whose enzymatic pathway has not been revealed in detail. As an alternative, I additionally propose an approach to obtain biosynthetically informed distance *d*_*A,B*_, which requires knowledge about the chemical class of a compound, but not the enzymes involved in its biosynthesis (see above). The lower resolution of the approach relying on chemical classes instead of enzymes can be compensated by merging the biosynthetically informed distances with Bray–Curtis dissimilarities, which results in astonishingly similar distances compared to those obtained from trials using information of the enzymes involved in the synthesis of the compounds (see Fig. 2).

As mentioned earlier, biosynthetically informed distance *d*_*A,B*_ may be irrelevant in studies where the similarity in secondary metabolite profiles is related to similarity in animal behavior toward these profiles. Animals often respond to key compounds or a number of compounds in defined ratios (Junker et al. 2017; Bruce et al. 2005), but certainly not to the presence or absence of a biosynthetic pathway. Therefore, the research question should dictate which distance measure is best suited. Research questions that may benefit the most of the biosynthetically informed distance *d*_*A,B*_ are discussed below.

### Concluding remarks

Conventional distance measures applied for secondary metabolite profiles do not consider the biosynthetic pathways of the compounds (Brückner and Heethoff 2017). Thereby, the reciprocal dependence of compounds due to their shared biosynthetic pathways is ignored, which clearly affects the production of secondary metabolites (Junker et al. 2017). The approach proposed here directly addresses this concern and calculates biosynthetically informed distances between secondary metabolite profiles and consequently considers the similarity of the biosynthetic pathways of the compounds additionally to their identity. Next to information on biosynthesis, the approach uses generalized UniFrac distances (Chen et al. 2012; Lozupone and Knight 2005) that have been developed to calculate the distance between microbial communities and is now a standard approach in microbiome research.

In general, biosynthetically informed distances represent a useful addition to conventional distance measures commonly used in studies comparing secondary metabolite profiles and provide information about the similarity of the enzymatic equipment of the organisms that have been sampled rather than the similarity between the compositions of the compounds detected. In my opinion, the new approach mainly benefits studies in community ecology and in studies searching for phylogenetic signals in secondary metabolite profiles. Community ecology will produce data sets comprising the secondary metabolite profiles of multiple species but with few replicates per species. Such a dataset will comprise a large proportion of compounds that are present in one or few species (and thus samples) only (Junker 2016), which will limit the validity of interpretations of results based on conventional distance measures. By considering the biosynthetic pathways leading to the compounds instead of exclusively the identity of the compounds, the functional composition and diversity of a community may be assessed in a more meaningful way. For phylogenetic investigations, the approach may likewise yield more meaningful results. In conventional distance measures, the addition of any new compound is considered in exactly the same way regardless the biosynthetic origin. Let us assume two species that differ in a single compound only: one species contains α-pinene and the other one, e.g., β-phellandrene (both monoterpenes, see Fig. 1), both species have nearly the same enzymatic equipment that differs only in the final enzyme of the pathway. In contrast, if one species contains α-pinene and the other one indole (aromatic compound, Fig. 1), the species possess completely different pathways. Despite these differences, conventional distance measure would return the same distances although the former example indicates a conserved (or converged, depending on the phylogenetic distance of the species) evolutionary relationship and the latter a diverged evolutionary relationship if biosynthetic information is considered. These strong differences in the assessment of the evolutionary relationship of two species would be precisely depicted using biosynthetically informed distances *d*_*A,B*_. The main and the alternative approaches introduced here are highly flexible in incorporating any information on the compounds that is of relevance in a given study. For instance, instead of information on the enzymes involved in the biosynthesis of compounds or their chemical class, functional groups, other chemical properties, or even ecological functions can be used to characterize the compounds. Therefore, biosynthetically informed distances *d*_*A,B*_ are broadly applicable and can be adopted to specific research questions.

Biosynthetically informed distances thus provide a novel way to compare secondary metabolite profiles considering the biosynthesis of the compounds present in the samples. This information is meaningful addition to conventional distance measures and allows novel insights into the functional composition and diversity of communities and the evolution of secondary metabolites.

## Electronic supplementary material

Below is the link to the electronic supplementary material.

Supplementary material 1 (ZIP 51 KB)
